# Dietary crude protein reductions in wheat-based diets with two energy densities compromised performance of broiler chickens from 15 to 36 days post-hatch

**DOI:** 10.1016/j.psj.2023.102932

**Published:** 2023-07-16

**Authors:** Shemil P. Macelline, Peter V. Chrystal, Mehdi Toghyani, Peter H. Selle, Sonia Y. Liu

**Affiliations:** ⁎ School of Life and Environmental Sciences, Faculty of Science, The University of Sydney, Sydney NSW 2006, Australia; † Poultry Research Foundation, The University of Sydney, Camden NSW 2570, Australia; # Complete Feed Solutions, Hornsby NSW 2071, Australia; Howick 2145, New Zealand; ‡ Sydney School of Veterinary Science, The University of Sydney, Sydney NSW 2006, Australia

**Keywords:** ammonia, broiler, energy, protein, wheat

## Abstract

This study was designed to investigate the impacts of 2 energy densities (13.0 and 12.5 MJ/kg ME) in wheat-based diets with 3 tiers of CP concentrations (210, 190, and 170 g/kg) on the performance of broiler chickens. The parameters assessed included growth performance (15–36 d posthatch), carcass traits, nutrient utilization, starch–protein digestive dynamics, apparent ileal amino acid digestibility coefficients, and the free amino acid and ammonia (**NH_3_**) concentrations in systemic plasma. Also, the feasibility of substituting soybean meal with canola meal in 190 g/kg CP diets was investigated. The dietary CP reduction from 210 to 170 g/kg significantly compromised weight gain by 12.4% (1,890 vs. 2158 g/bird) and FCR by 5.33% (1.501 vs. 1.425). The 0.5 MJ energy density reduction compromised FCR by 3.25% (1.525 vs. 1.477; *P* = 0.013) in birds offered 170 g/kg CP diets. Reducing dietary CP and energy densities interactively influenced (*P* = 0.027) apparent metabolizable energy (**AME**) and nitrogen corrected metabolizable energy (**AMEn**) (*P* = 0.022) such that reducing dietary CP increased these parameters but reducing dietary energy densities decreased AME and AMEn. The 150 g/kg canola meal inclusion with the elimination of soybean meal displayed some promise. Dietary CP reductions (and increased nonbound amino acid inclusions) linearly associated with increased plasma ammonia (NH_3_) concentrations (r = −0.607; *P* = 0.010) and plasma NH_3_ was linearly related to depressed weight gains (r = −0.565; *P* = 0.018). The association of dietary non–protein-bound amino acid (**NPBAA**) inclusions and elevated plasma NH_3_ concentrations have profound implications for the successful development of reduced-CP, wheat-based broiler diets.

## INTRODUCTION

The successful development of wheat-based, reduced-crude protein (**CP**) diets is proving to be a real challenge (Selle et al., 2023a), which is of obvious relevance to all countries, including Australia, where wheat is the dominant feed grain for chicken-meat production. However, dietary CP reductions in maize-based diets have been shown to improve apparent metabolizable energy (**AME**) in broiler chickens across 4 similar studies (Chrystal et al., 2020a,b,c; 2021). Collectively, CP reductions from an average of 210 to 163 g/kg increased AME by 0.34 MJ (13.16 vs. 12.82 MJ/kg) in these 4 studies. The increased AME in reduced-CP broiler diets may have stemmed from diminished thermogenesis or heat increment from lower protein intakes (Musharaf and Latshaw, 1999). In broad terms, the cost of 1 MJ of energy density in a 13.0 MJ/kg broiler diets is in the order of US$ 31.00 per tonne under Australian conditions, but considerably more when energy is derived solely from tallow or vegetable oil. Clearly, it would be beneficial for chicken-meat production if advantage could be taken of this apparent energy-sparing effect in reduced-CP diets.

However, the impact of wheat-based, reduced-CP diets on energy utilization is not equally straightforward. The CP reduction from 215 to 165 g/kg numerically depressed AME by 0.25 MJ (11.52 vs. 11.77 MJ/kg) and significantly compromised AMEn by 0.41 MJ (10.87 vs. 11.28 MJ/kg; *P* = 0.027) in Yin et al. (2020). It does appear that broiler chickens can better accommodate CP reductions in maize- than wheat-based diets, which was evident in Chrystal et al. (2021). The reasons for this difference are almost certainly complex and may include more rapid starch digestion rates (Giuberti et al., 2012) and higher concentrations of soluble nonstarch polysaccharides in wheat (Bach Knudsen, 1997) coupled with higher protein concentrations in wheat compared to maize (Selle et al., 2022). Wheat's higher protein concentrations necessitate higher inclusions of non–protein-bound (synthetic, crystalline) amino acids (**NPBAA**) in reduced-CP diets to meet specifications, but there are probably limits to the extent that protein-bound amino acids can be replaced by NPBAA before growth performance is compromised (Baker, 2009).

Nevertheless, reduced-CP diets hold several potential advantages including attenuated nitrogen (**N**) and ammonia (**NH_3_**) emissions, better litter quality, bird welfare, and flock health (Greenhalgh et al., 2020a). Additionally, reduced-CP diets demand lower soybean meal inclusions, which is an expensive, imported feedstuff for most countries in the world. Australian canola production is forecast to reach a record high of 8.3 million tonnes in 2022–23 (ABARES, 2023), much of which is exported. Thus, canola meal is a potential alternative to soybean meal for Australian chicken-meat production, but its present usage is quite often limited to dietary inclusions of less than 100 g/kg.

Therefore, the present study was designed to investigate the impacts of 2 dietary energy densities (13.0 and 12.5 MJ/kg ME) in wheat-based diets with 3 tiers of dietary CP concentrations (210, 190, and 170 g/kg) on the performance of broiler chickens from 15 to 36 d posthatch to assess the feasibility of decreasing energy densities by 0.5 MJ in reduced-CP, wheat-based diets. The performance parameters assessed included growth performance, relative fat-pad weights, carcass traits, nutrient utilization, starch–protein digestive dynamics, apparent ileal amino acid digestibility coefficients, free amino acid, and NH_3_ concentrations in systemic plasma. Additionally, the feasibility of substituting soybean meal with canola meal in reduced-CP diets was evaluated.

## MATERIALS AND METHODS

### Animal Ethics

All experimental procedures fully complied with specific guidelines (2019/1516) issued by the Animal Ethics Committee of the University of Sydney.

### Experimental Design

The experimental design comprised a 2 × 3 + 1 array of dietary treatments. The 2 × 3 factorial design comprised 3 dietary crude protein concentrations (210, 190, and 170 g/kg) and 2 dietary apparent metabolizable energy densities of 13.0 and 12.5 MJ/kg. The additional treatment was included to determine the effect of canola meal inclusions as a substitute for soybean meal in 190 g/kg CP diets. The arrangement of dietary treatments is presented in Table 1. The composition and nutrient specifications of the experimental diets are shown in Tables 2 and 3, respectively.

**Table 1:** Arrangement of dietary treatments.

Treatments	Dietary CP (g/kg)	Dietary AME (MJ/kg)	Canola meal inclusions (g/kg)
1A	210	13.0	0
2B	190	13.0	0
3C	170	13.0	0
4D	210	12.5	0
5E	190	12.5	0
6F	170	12.5	0
7G	190	13.0	150

Abbreviation: AME, apparent metabolizable energy.

**Table 2:** Composition of experimental diets.

Item (g/kg)	1A	2B	3C	4D	5E	6F	7G
Wheat (156 g/kg)	738	844	737	755	796	739	617
Maize starch	10.8	-	103	-	-	68.1	100
Canola meal (386 g/kg)	-	-	-	-	-	-	150
Soybean meal (505 g/kg)	156	37.4	-	150	56.8	-	-
*d,l* – Methionine	2.71	3.60	4.59	2.70	3.61	4.58	3.10
Glycine	0.01	3.54	6.19	0.08	3.31	6.17	2.79
*l*-Arginine	2.29	5.83	7.78	2.41	5.44	7.77	5.42
*l*-Histidine	-	0.62	1.39	-	0.54	1.38	0.41
*l*-Isoleucine	0.89	2.77	4.06	0.94	2.62	4.05	2.78
*l*-Leucine	-	3.08	5.32	0.07	2.86	5.30	2.92
*l*-Lysine HCl	5.64	9.32	11.0	5.80	8.84	11.03	8.15
*l*-Phenylalanine	0.84	4.62	6.96	0.96	4.26	6.95	4.84
*l*-Threonine	2.67	4.23	5.22	2.72	4.09	5.22	3.72
*l*-Tryptophan	-	0.33	0.71	-	0.28	0.71	0.22
*l*-Valine	1.26	3.14	4.56	1.30	3.02	4.55	2.81
Soy oil	28.9	15.0	15.0	15.00	15.0	15.0	36.8
Limestone	14.4	14.9	14.9	14.4	14.7	14.9	13.3
Monocalcium P	6.40	7.32	8.24	6.40	7.32	8.25	4.30
Potassium carbonate	0.82	5.09	7.12	1.00	4.54	7.11	4.54
Sodium bicarbonate	4.28	4.31	4.40	4.27	4.32	4.40	3.62
Vit–min premix^1^	2.20	2.20	2.20	2.20	2.20	2.20	2.20
Choline Cl (60%)	1.00	1.00	1.00	1.00	1.00	1.00	1.00
Phytase^2^	0.10	0.10	0.10	0.10	0.10	0.10	0.10
Xylanase^3^	0.20	0.20	0.20	0.20	0.20	0.20	0.20
Celite	20.0	20.0	20.0	20.0	20.0	20.0	20.0
Inert filler (sand)	-	6.93	28.8	13.2	39.0	62.3	9.85
Total NPBAA	15.1	39.0	55.4	15.7	36.9	55.3	35.4

1 Vitamin-trace mineral premix supplies in MIU/kg or mg/kg of diet: [MIU] retinol 12, cholecalciferol 5, [mg] tocopherol 50, menadione 3, thiamine 3, riboflavin 9, pyridoxine 5, cobalamin 0.025, niacin 50, pantothenate 18, folate 2, biotin 0.2, copper 20, iron 40, manganese 110, cobalt 0.25, iodine 1, molybdenum 2, zinc 90,selenium 0.3.

2 phytase (Axtra PHY, Dupont Nutrition & Bioscience) 10,000 FTU.

3 xylanase (Danisco, Dupont Nutrition & Bioscience) 40,000 U/g.

**Table 3:** Nutrient specifications of experimental diets (as-is basis).

Nutrient (g/kg)	1A	2B	3C	4D	5E	6F	7G
Dry matter	912	916	929	911	918	929	924
AME, MJ/kg	13.0	13.0	13.0	12.5	12.5	12.5	13.0
Crude protein	210	190	170	210	190	170	190
Starch	471	526	560	471	495	526	482
Lysine^1^	11.0	11.0	11.0	11.0	11.0	11.0	11.0
Methionine	5.13	5.52	6.05	5.12	5.55	6.04	5.33
TSAA	8.14	8.14	8.14	8.14	8.14	8.14	8.14
Threonine	7.70	7.70	7.70	7.70	7.70	7.70	7.70
Valine	8.69	8.69	8.69	8.69	8.69	8.69	8.69
Isoleucine	7.59	7.59	7.59	7.59	7.59	7.59	7.59
Leucine	11.8	11.8	11.8	11.8	11.8	11.8	11.8
Arginine	12.1	12.1	12.1	12.1	12.1	12.1	12.1
Histidine	4.06	3.63	3.63	4.04	3.63	3.63	3.63
Tryptophan	2.00	1.76	1.76	1.99	1.76	1.76	1.76
Glycine_equivalent_^2^	12.3	12.9	13.3	12.3	12.8	13.3	12.3
Phenylalanine	8.19	6.13	4.61	8.15	6.27	4.62	5.86
Phe + tyrosine	12.8	12.8	12.8	12.8	12.8	12.8	12.8
Calcium	8.70	8.70	8.70	8.70	8.70	8.70	8.70
Available P	4.35	4.35	4.35	4.35	4.35	4.35	4.35
Crude fiber	15.4	14.4	12.0	15.5	14.0	11.9	25.5
Crude fat	46.2	33.0	30.3	32.8	32.3	30.3	54.7
DEB^3^	210	210	210	210	210	210	210

1 All amino acids are expressed on standardized ileal digestible basis.

2 Glycine equivalent = glycine concentration + [serine concentration × 0.7143].

3 DEB: dietary electrolyte balance = K^+^ + Na^+^-Cl^−^.

### Diet Preparation

The diets were formulated based on near-infrared spectroscopy (**NIR**) of wheat, soybean meal, and canola meal via the AMINONir Advanced program (Evonik Nutrition & Care GmbH, Hanau, Germany). The experimental diets were formulated to contain 11.0 g/kg standardized digestible lysine, 8.14 g/kg of standardized digestible sulfur amino acids, and 7.70 g/kg of standardized digestible threonine concentrations. All diets contained a xylanase (Danisco Animal Nutrition, København, Denmark) and phytase (Axtra Phy, Danisco Animal Nutrition) feed enzymes. Acid insoluble ash (Celite World Minerals, Lompoc, CA) was included at 20 g/kg in all diets as an inert marker to determine the digestibility coefficients of starch, protein (N), and amino acids. The diets were steam-pelleted at a conditioning temperature of 80°C with a residence time of 14 s. Dietary energy densities were adjusted by changing either wheat or maize starch or soy oil concentrations to achieve targeted energy densities.

### Bird Management

A total of 252 off-sex (parent line), male Ross 308-day-old chicks were procured from a commercial hatchery and offered a common starter diet from 1 to 14 d posthatch. At 15 d posthatch, birds were individually identified (wing-tags) and allocated into 42 cages (6 birds per pen) based on body weights. The mean body weight of the 42 cages was 482 ± 4.1 g/bird at 15 d posthatch. Each of the dietary treatments was then offered to 6 replicate battery cages, from 15 to 36 d posthatch. The cage dimensions were 750 × 750 × 500 mm in width, length, and height. Birds had unrestricted access to feed and water in an environmentally controlled facility with 23-h-on-1-h-off lighting regime for the first week and 18-h-on-6-h-off lighting regime during wk 1 to 5. An initial room temperature of 32 ± 1°C was maintained for the first week, which was gradually decreased to 22 ± 1°C by the end of the third week and maintained at this temperature for the duration of the feeding study.

### Data and Sample Collection, Chemical Analyses, and Calculations

Growth performance (weight again, feed intake, feed conversion ratio [FCR]) was determined from 15 to 36 d posthatch. Birds were weighed at d 15 and 36 and feed intakes were monitored over this interval, bodyweights of any dead or culled birds were recorded daily to correct feed intakes on a per cage basis to adjust FCR calculations.

Total excreta output and feed intake were monitored from 27 to 29 d posthatch to determine energy and crude protein utilizations that includes AME, AME to gross energy (**GE**) ratio, N retention, and AMEn. Wet excreta weight was taken prior to drying in a forced-air oven at 80°C for 24 h to determine excreta dry matter. The GE of excreta and diets were determined using a Parr 1281 adiabatic bomb calorimeter (Parr Instrument CO., Moline, IL) to calculate AME values of the diets on dry matter basis as following equation:

$$\text{AM}E_{\text{diet}}\;\left(\text{MJ}/\text{kg}\right)\;=\left[\text{feed}\;\text{intake}\;\left(\text{kg}\right)\;\times \;GE_{\text{diet}}\;\left(\text{MJ}/\text{kg}\right)\right]-\left[\text{excreta}\;\text{output}\;\left(\text{kg}\right)\;\times GE_{\text{excreta}}\left(\text{MJ}/\text{kg}\right)\right]$$

The AME:GE ratios were calculated by dividing AME by the GE in respective diets.

The N contents of diets and excreta were determined using a nitrogen determinator (Leco Corporation, St Joseph, MI) and N retentions in each diet calculated from the following equation:

$$N\;\text{retention}\;\left(\%\right)\;=\;\frac{\left[\text{feed}\;\text{inatake}\;\left(\text{kg}\right)\;\times \;N-\text{diet}\;\left(\%\right)\right]\;-\;\left[\text{excreta}\;\left(\text{kg}\right)\;\times \;N-\text{excreta}\;\left(\%\right)\right]}{\left[\text{feed}\;\text{inatake}\;\left(\text{kg}\right)\;\times \;N-\text{diet}\;\left(\%\right)\right]}\;\times \;100$$

The AMEn values were calculated by correcting N retention to zero using the factor of 36.54 kJ/g N retained in the body (Hill and Anderson, 1958).

At 34 d posthatch, blood samples were taken from the brachial vein of 3 representative birds per cage to determine the concentrations of free amino acid and ammonia in systemic plasma. Blood samples were centrifuged, and decanted plasma samples were held at −80°C prior to analysis. Concentrations of 20 proteinogenic amino acids and ammonia were determined using precolumn derivatization amino acid analysis with 6-aminoquinolyl-N-hydroxysuccinimidyl carbamate (AQC; Waters AccQTag Ultra; Waters Australia PL; www.waters.com) followed by separation of the derivatives and quantification by reversed phase ultra-performance liquid chromatography (**RP-UPLC**; Waters Australia, Rydalmere, Australia). All amino acids were detected by UV absorbance and this procedure is fully described in Selle et al. (2016).

At d 36, all the birds from each cage were euthanized by intravenous injections of sodium pentobarbitone, the abdominal cavity opened, and small intestines removed. Digesta samples were collected in their entirety from the distal jejunum and distal ileum. The distal jejunum was demarcated by the mid-point between the end of the duodenal loop and Meckel's diverticulum and the distal ileum was demarcated by the mid-point between Meckel's diverticulum proximally and the ileo-caecal junction distally. Digesta were manually expressed from this segment and digesta samples were pooled for each pen, homogenized, freeze-dried, and ground through 0.5 mm screen to analyze starch, protein (N), and amino acids concentrations. In addition, abdominal fat-pads dissected out, weighed and recorded against final body weights to calculate relative abdominal fat-pad weights. Also, *Pectoralis* major, *Pectoralis* minor, and thigh muscles were removed from the carcass and recorded against final body weights to calculate relative weights of carcass traits.

Starch concentrations in feed and digesta samples were determined by using total starch assay kits (Megazyme, Wicklow, Ireland) as described in Mahasukhonthachat et al. (2010). Nitrogen contents of diets and digesta were determined using a nitrogen determinator (Leco Corporation) by the Dumas method and AIA concentrations were determined by the method described by Siriwan et al. (1994). Amino acid concentrations of diets and digesta were determined by 24-h liquid hydrolysis at 110°C in 6 M HCl followed by analysis of 16 amino acids using the Waters AccQTag Ultra chemistry on a Waters Acquity UPLC (Waters Corporation, Milford, MA). The apparent digestibility coefficients for starch, protein (N), and amino acids sites were calculated from the following equation:

$$\text{Digestibility}\;\text{coefficient}=\;\frac{{\left(\text{Nutrient}/\text{AIA}\right)}_{\text{diet}}-\;{\left(\text{Nutrient}/\text{AIA}\right)}_{\text{digesta}}}{{\left(\text{Nutrient}/\text{AIA}\right)}_{\text{diet}}}$$

Starch, protein (N), and amino acid disappearance rates (g/bird/d) were calculated from the following equation:

$$\text{Nutrient}\;\text{disappearance}\;\text{rat}e_{\left(g/\text{bird}/d\right)}=\text{feed}\;\text{intak}e_{\left(g/\text{bird}\right)}\times \text{dietary}\;\text{nutrien}t_{\left(g/\text{kg}\right)}\times \text{digestibility}\;\text{coefficient}.$$

Ratios of starch to protein disappearance rates in the distal ileum were calculated as this eliminates the confounding influence of feed intake.

### Statistical Analyses

The experimental data were analyzed by 2-way analyses of variance using the JMP Pro 16.0 software package (SAS Institute Inc. JMP Software, Cary, NC). Pair-wise comparisons were performed between diet 2B and 7G. Linear and quadratic relationships and multiple linear regressions were established when considered appropriate. Pen means were the experimental unit and a probability level of less than 5% was considered statistically significant.

## RESULTS

The effect of dietary treatments on growth performance and relative fat-pad weights are shown in Table 5. Dietary CP reductions significantly (*P* < 0.001) depressed weight gains by up to 12.4% (1,890 vs. 2,158 g/bird), feed intakes by up to 7.19% (2,852 vs. 3,073 g/bird), and compromised FCR by up to 5.33% (1.501 vs. 1.425). The overall mortality was 1.98% (data not shown) and not related to treatments (*P* = 0.915). Relative fat-pad weights were significantly increased by 47.4% (8.68 vs. 5.89 g/kg/bird) when the 210 and 170 g/kg CP diets are compared. The 0.5 MJ reduction in energy density compromised FCR by an average of 1.65% (1.477 vs. 1.453; *P* = 0.026). The substitution of soybean meal with canola meal did not significantly influence growth performance and relative fat-pad weights.

The relative weights or yields of *Pectoralis major, Pectoralis minor*, and leg shanks at 36 d posthatch are shown in Table 6. There were no significant treatment effects on carcass traits including the substitution of soybean meal with canola meal.

The effects of dietary treatments on nutrient utilization are displayed in Table 7. A treatment interaction (*P* = 0.027) was observed for AME as the energy density reduction depressed AME by 0.73 MJ (13.88 vs. 14.62 MJ/kg) in 210 g/kg CP diets and by 0.53 MJ (14.46 vs. 14.99 MJ/kg) in 170 g/kg CP diets, but no difference was observed in birds offered 190 g/kg CP diets. A treatment interaction (*P* = 0.014) effect also was found for AME:GE ratios as energy density reductions improved AME:GE ratio by 3.55% (0.817 vs. 0.789) in 190 g/kg CP diets but did not influence efficiency of energy utilization in birds offered 210 and 170 g/kg CP diets. Reducing dietary CP significantly (*P* < 0.001) enhanced N retention by up to 8.20 percentage units (73.8 vs. 65.6%). There was a significant treatment interaction (*P* = 0.022) for AMEn as the energy density reduction significantly increased AMEn by 0.11 MJ in 190 g/kg CP diets, but significantly decreased AMEn by 0.55 and 0.59 MJ in the 210 and 170 g/kg CP diets, respectively. The substitution of soybean meal with canola meal improved AME by 0.55 MJ (14.96 vs. 14.41 MJ/kg; *P* = 0.002) and AMEn by 0.66 MJ (13.63 vs. 12.97 MJ/kg; *P* = 0.001) in 190 g/kg CP diets.

Table 8 shows treatment effects on apparent protein (N) digestibility coefficients and disappearance rates in distal jejunum and distal ileum. Protein (N) digestibility coefficients were not significantly influenced by treatment in both intestinal segments. However, dietary CP reductions significantly retarded (*P* < 0.001) distal jejunal protein (N) disappearance rates by up to 21.3% (17.50 vs. 22.23 g/bird/d; *P* < 0.001). A treatment interaction (*P* = 0.009) was observed for distal ileal protein (N) disappearance rates as energy density reductions retarded disappearance rates by 5.10% (24.77 vs. 26.10 g/bird/d) in 210 g/kg CP diets, whereas energy density reductions accelerated disappearance rates by 7.23% (20.03 vs. 18.68 g/bird/d) in birds offered 170 g/kg CP diets. The substitution of soybean meal with canola meal retarded protein (N) distal jejunal disappearance rates by 13.4% (18.86 vs. 21.79 g/bird/d; *P* = 0.002) and distal ileal disappearance rates by 2.37% (22.67 vs. 23.22 g/bird/d; *P* < 0.001).

The effects of dietary treatments on starch digestibility coefficients, disappearance rates, and starch:protein disappearance rate ratios in distal jejunum and distal ileum are shown in Table 9. Significant treatment interactions were observed for all the assessed parameters. The treatment interaction for distal jejunal starch digestibility coefficients in distal jejunum (*P* = 0.047) was because energy density reductions tended to decrease starch digestibility in birds offered 210 g/kg CP diets but has the opposite trend in 190 and 170 g/kg CP diets. The treatment interaction (*P* = 0.009) for distal ileal starch digestibility coefficients stemmed from a similar pattern of responses but with a small, but significant increase in starch digestibility in birds offered 190 g/kg CP diets with reduced energy density. Treatment interactions for starch disappearance rates were observed in distal jejunum (*P* = 0.013) and distal ileum (*P* = 0.007). This was because, in both segments, the energy density reduction significantly retarded starch disappearance rates in birds offered 190 g/kg CP diets by 8.45% (64.36 vs. 70.30 g/bird/d) in the jejunum and by 9.44% (67.53 vs. 74.57 g/bird/d) in the ileum. In contrast, the energy density reduction had no significant influence in 210 and 170 g/kg CP diets in both segments. There was a treatment interaction (*P* = 0.014) for starch:protein disappearance rate ratios in the distal jejunum because energy density reductions significantly increased disappearance rate ratios from 2.74 to 3.03, but only in 210 g/kg CP diets by 10.6% (3.03 vs. 2.74). A treatment interaction (*P* < 0.001) was also observed in the distal ileum where starch:protein disappearance rate ratios were significantly increased by energy density reductions in birds offered 210 g/kg CP diets, but significantly decreased when birds were offered both 190 and 170 g/kg CP diets, The substitution of soybean meal with canola meal increased distal jejunal starch digestibility by 2.79% (0.959 vs. 0.933; *P* = 0.002), significantly retarded starch disappearance rates in both the jejunum and ileum and condensed starch:protein disappearance rate ratios from 3.21 to 2.98 (*P* = 0.005) in the distal ileum.

The effects of dietary treatments on apparent ileal digestibility coefficients of essential amino acids are shown in Table 10, where significant responses are limited. Dietary CP reductions depressed histidine digestibility by 3.96% (0.801 vs. 0.834; *P* = 0.026) and energy density reductions enhanced methionine digestibility by 1.38% (0.956 vs. 0.943; *P* = 0.007). The canola meal substitution increased methionine digestibility by 2.14% (0.955 vs. 0.935; *P* = 0.017). The outcomes for nonessential amino acid digestibility coefficients are presented in Table 11. Dietary CP reductions depressed digestibility coefficients of alanine by 13.1% (678 vs. 0.780; *P* = 0.001), aspartic acid by 19.1% (0.649 vs. 0.802; *P* < 0.001), glutamic acid by 2.08% (0.898 vs. 0.917), and serine by 7.94% (0.777 vs. 0.844; *P* < 0.001), whereas dietary CP reductions increased glycine digestibility coefficient by 5.20% (0.849 vs. 0.807; *P* < 0.001). Energy density reductions increased digestibility of alanine by 5.82% (0.746 vs. 0.705; *P* = 0.035) and aspartic acid by 5.81 (0.747 vs. 0.706). The canola meal substitution depressed proline digestibility by 3.33% (0.870 vs. 0.900; *P* = 0.004).

Treatment effects on free amino acid and ammonia concentrations in systemic plasma in diets with an energy density of 13.0 MJ are shown in Table 12. Dietary CP reductions from 210 to 170 g/kg significantly increased plasma concentrations of methionine by up to 75.2% (28.2 vs. 16.1 µg/g), threonine by up to 37.6% (123.4 vs. 89.7 µg/g), glycine by up to 78.9% (68.7 vs. 38.4 µg/g), and tyrosine by up to 39.2% (52.6 vs. 37.8 µg/g). Conversely, dietary CP reductions decreased plasma concentrations of alanine by up to 35.5% (41.1 vs. 63.7 µg/g), asparagine by up to 45.9% (17.0 vs. 31.4 µg/g), aspartic acid by up to 31.9% (2.46 vs. 3.61), and cysteine by up to 19.7% (11.0 vs. 13.7). The linear effects of dietary CP reductions on free plasma amino acid concentrations are also tabulated. Dietary CP reductions significantly increased (*P* = 0.004) plasma NH_3_ concentrations (determined as NH_4_OH) by up to 17.1% (2.19 vs. 1.87 µg/g) and the linear effect was significant (r = −0.607; *P* = 0.010).

## DISCUSSION

Broiler growth performance in the present study was satisfactory in that birds offered 210 g/kg CP diets that exceeded 2022 Aviagen performance objectives for male Ross 308 broilers by 10.7% (2,158 vs. 1,949 g/bird) in weight gain and by 6.68% (1.425 vs. 1.527) in FCR. Nevertheless, the dietary CP reduction from 210 to 170 g/kg compromised weight gain by 12.4% (1,890 vs. 2,158 g/bird) and FCR by 5.33% (1.501 vs. 1.425), which is not acceptable. This outcome epitomizes the challenge to reduce CP concentrations in wheat-based broiler diets. Across 7 Australian feeding studies, reduced-CP, wheat-based diets compromised median responses in weight gain by 9.17% and in FCR by 6.30% (Selle et al., 2023a), which are quite consistent with the outcomes of the present study. Moreover, it has been demonstrated in 2 direct comparisons (Chrystal et al., 2021; Greenhalgh et al., 2022a) that birds offered maize-based diets are better able to accommodate dietary CP reductions than wheat-based diets. One of the underlying reasons for this difference appears to be the higher protein contents of wheat than maize. Wheat contained 115.5 g/kg CP as opposed to 80.0 g/kg in maize in one Australian survey (Bryden et al., 2009). The higher wheat protein concentration means that elevated dietary NPBAA inclusions are required to meet specifications. However, wheat used in the present study contained 156 g/kg CP; consequently the 210, 190, and 170 g/kg CP diets contained an average of 15.4, 38.0, and 55.4 g/kg NPBAA (Table 2), respectively. These are relatively high inclusion levels and may have triggered postenteral amino acid imbalances (Selle et al., 2023b). Any amino acid imbalances will lead to the deamination of surplus amino acids and the generation of ammonia. Therefore, it appears that dietary CP reductions (and elevated NPBAA inclusions) linearly associated with increased plasma ammonia concentrations (r = −0.607; *P* = 0.010). Moreover, plasma ammonia concentrations were linearly associated with depressions in weight gain (r = −0.565; *P* = 0.018) and feed intake (r = −0.487; *P* = 0.048), which suggests inadequate ammonia detoxification may have contributed to compromised growth performance. Similar relationships between plasma ammonia concentrations and broiler growth performance have been reported (Namroud et al., 2008; Ospina-Rojas et al., 2013, 2014). Glycine supplementation maintained analyzed glycine equivalent concentrations at an average of 14.15 g/kg across all dietary treatments in the present study (Table 4). Glycine (and serine) are prerequisites for the Krebs uric acid cycle, which eliminates NH_3_-N as uric acid-N (Salway, 2018). The likelihood is that this maintenance of glycine equivalents prevented pronounced growth performance deterioration in birds offered reduced-CP, wheat-based diets as reported by Greenhalgh et al. (2020b) and Chrystal et al. (2021).

**Table 4:** Analyzed nutrient compositions of experimental diets (as-is basis).

Item (g/kg)	1A	2B	3C	4D	5E	6F	7G
Dry matter	887	891	906	891	887	903	885
Gross energy (MJ/Kg)	16.61	16.27	16.07	16.20	15.68	15.66	16.63
Crude protein	211	193	173	203	189	172	182
Starch	454	518	554	470	463	511	446
Starch:protein ratio	2.15	2.68	3.20	2.32	2.45	2.97	2.45
Arginine	13.0	11.9	12.2	12.4	12.0	11.6	11.5
Histidine	4.68	3.83	3.72	4.50	3.87	3.75	3.79
Isoleucine	8.84	8.08	7.96	8.44	8.10	7.77	8.00
Leucine	14.1	13.0	12.9	13.5	12.9	12.7	12.7
Lysine	11.9	11.0	11.1	11.6	11.1	10.6	10.9
Methionine	4.78	5.23	5.98	4.92	5.21	5.29	4.87
Phenylalanine	10.2	9.44	9.54	9.70	9.47	9.31	9.10
Threonine	8.89	8.06	8.13	8.63	8.20	7.77	8.20
Valine	10.2	9.70	9.60	9.87	9.48	9.39	9.57
Alanine	7.26	5.17	3.90	6.96	5.29	3.94	5.50
Aspartic acid	15.3	8.75	5.74	14.3	13.8	5.85	8.47
Glutamic acid	51.7	44.3	36.9	50.4	43.5	37.7	40.0
Glycine	8.30	9.66	10.7	8.11	9.33	10.2	9.17
Proline	15.9	14.2	12.0	15.6	13.8	12.2	13.1
Serine	9.67	7.12	5.54	9.30	7.20	5.70	6.84
Tyrosine	4.74	4.43	4.37	4.60	4.52	4.29	4.48
Total amino acids	199	174	160	193	174	158	166
Glycine equivalents^1^	15.21	14.75	14.66	14.75	11.35	14.27	14.06

1 Glycine equivalent = glycine concentration + [serine concentration × 0.7143].

**Table 5:** The effect of dietary treatments on growth performance in broiler chickens from 15 to 36 d posthatch and relative abdominal fat-pad weights at 36 d posthatch.

Diet	Treatment		Growth performance			Relative abdominal fat-pad weight (g/kg)
	CP (g/kg)	Energy (MJ/kg)	Weight gain (g/bird)	Feed intake (g/bird)	FCR (g/g)	
1A	210	13.0	2,171	3,064	1.411	6.53
2B		12.5	2,144	3,082	1.439	5.25
3C	190	13.0	2,078	3,057	1.470	6.41
4D		12.5	2,091	3,071	1.468	7.15
5E	170	13.0	1,880	2,777	1.477	9.08
6F		12.5	1,919	2,926	1.525	8.28
SEM						
Main effect: crude protein			34.9	54.3	0.0129	0.595
210			2,158^c^	3,073^b^	1.425^a^	5.89^a^
190			2,085^b^	3,064^b^	1.469^b^	6.78^a^
170			1,890^a^	2,852^a^	1.501^c^	8.68^b^
Energy density						
13.0			2043	2966	1.453^a^	7.34
12.5			2052	3026	1.477^b^	6.90
Significance (*P* =)						
Crude protein			<0.001	<0.001	<0.001	< 0.001
Energy density			0.771	0.184	0.026	0.369
Crude protein × energy			0.637	0.375	0.167	0.228
7G (150 g//kg canola meal, 190 g/kg CP)						
Pair-wise comparison (*P* =)			2,030	2,945	1.450	7.77
2B vs. 7G			0.277	0.130	0.241	0.106

a,b,c Means within columns not sharing a common superscript are significantly different at the 5% level of probability.

**Table 6:** The effect of dietary treatments on absolute and relative weights of carcass traits at 36 d posthatch.

Diet	Treatment		Relative weights (g/kg)			
			*Pectoralis major*	*Pectoralis minor*	Total breast	Thigh
	Crude protein (g/kg)	Energy (MJ/kg)				
1A	210	13.0	203	37.3	241	205
4D		12.5	195	34.8	230	202
2B	190	13.0	193	35.3	229	214
5E		12.5	189	32.9	222	199
3C	170	13.0	187	34.5	221	210
6F		12.5	185	33.3	219	208
SEM			5.3	0.93	5.9	6.4
Main effect						
Crude protein (g/kg)						
210			199	36.0	235^b^	204
190			191	34.1	225^ab^	206
170			186	33.9	220^a^	209
Energy (MJ/kg)						
13.0			194	35.7^b^	230	210
12.5			190	33.7^a^	224	203
Significance						
Crude protein			0.054	0.053	0.040	0.721
Energy			0.308	0.012	0.184	0.211
Crude protein × energy			0.816	0.731	0.787	0.515
7G (150 g/kg CM with 190 g/kg CP)			186	33.9	220	212
Pair-wise comparisons (*P*-value)						
2B vs. 7G			0.402	0.383	0.374	0.866

a,b,c Means within columns not sharing a common superscript are significantly different at the 5% level of probability.

**Table 7:** The effects of dietary treatments on parameters of nutrient utilization from 27 to 29 d posthatch.

Diet	Treatment		AME (DM)	AME:GE	N retention (%)	AMEn (DM)
	Crude protein (g/kg)	Energy (MJ/kg)				
1A	210	13.0	14.62^bc^	0.780^ab^	67.4	13.08^b^
4D		12.5	13.88^a^	0.763^a^	63.7	12.53^a^
2B	190	13.0	14.41^b^	0.789^b^	70.4	12.97^b^
5E		12.5	14.45^b^	0.817^c^	68.9	13.08^b^
3C	170	13.0	14.99^c^	0.845^d^	73.9	13.75^c^
6F		12.5	14.46^b^	0.834^cd^	73.7	13.16^b^
SEM			0.141	0.0077	1.56	0.132
Main effect						
Crude protein (g/kg)						
210			14.25	0.772	65.6^a^	12.80
190			14.43	0.802	69.7^b^	13.03
170			14.73	0.839	73.8^c^	13.46
Energy (MJ/kg)						
13.0			14.67	0.805	70.6	13.27
12.5			14.26	0.804	68.8	12.92
Significance						
Crude protein			0.007	<0.001	<0.001	<0.001
Energy			0.001	0.977	0.173	0.035
Crude protein × energy			0.027	0.014	0.549	0.022
7G (150 g/kg CM with 190 g/kg CP)			14.96	0.796	71.19	13.63
Pair-wise comparisons (*P*-value)						
2B vs. 7G			0.002	0.403	0.679	<0.001

a,b,c Means within columns not sharing a common superscript are significantly different at the 5% level of probability.

**Table 8:** The effects of dietary treatments on apparent protein (N) digestibility coefficients, and disappearance rates in distal jejunum and distal ileum at 36 d posthatch.

Diet	Treatment		Digestibility coefficients		Disappearance rates (g/bird/d)	
	Crude protein (g/kg)	Energy (MJ/kg)	Distal jejunum	Distal ileum	Distal jejunum	Distal ileum
1A	210	13.0	0.751	0.848	23.04	26.10^e^
4D		12.5	0.717	0.832	21.41	24.77^d^
2B	190	13.0	0.775	0.827	21.79	23.22^c^
5E		12.5	0.766	0.837	21.15	23.11^c^
3C	170	13.0	0.765	0.817	17.51	18.68^a^
6F		12.5	0.732	0.836	17.50	20.03^b^
SEM			0.0197	0.0112	0.590	0.403
Main effect						
Crude protein (g/kg)						
210			0.734	0.840	22.23^b^	25.44
190			0.770	0.832	21.47^b^	23.16
170			0.748	0.826	17.50^a^	19.35
Energy (MJ/kg)						
13.0						
12.5			0.764	0.831	20.78	22.67
			0.738	0.835	20.02	22.64
Significance						
Crude protein			0.194	0.476	<0.001	<0.001
Energy			0.121	0.643	0.124	0.930
Crude protein × energy			0.776	0.257	0.394	0.009
7G (150 g/kg CM with 190 g/kg CP)			0.739	0.819	18.86	20.91
Pair-wise comparisons (P-value)						
2B vs. 7G			0.169	0.647	0.002	<0.001

a,bc Means within columns not sharing a common superscript are significantly different at the 5% level of probability.

**Table 9:** The effects of dietary treatments on starch digestibility coefficients, disappearance rates and starch-protein disappearance rate ratios in distal jejunum and distal ileum at 36 d posthatch.

Diet	Treatment		Digestibility coefficients		Disappearance rates (g/bird/d)		Disappearance rate ratios	
	Crude protein (g/kg)	Energy (MJ/kg)	Distal jejunum	Distal ileum	Distal jejunum	Distal ileum	Distal jejunum	Distal ileum
1A	210	13.0	0.950^ab^	0.997^b^	63.02^a^	66.17^a^	2.74^a^	2.53^a^
4D		12.5	0.933^a^	0.992^ab^	64.30^a^	68.39^ab^	3.03^b^	2.76^b^
2B	190	13.0	0.933^a^	0.989^a^	70.30^b^	74.57^c^	3.23^b^	3.21^d^
5E		12.5	0.950^ab^	0.996^b^	64.36^a^	67.53^ab^	3.05^b^	2.92^c^
3C	170	13.0	0.948^ab^	0.993^ab^	69.48^b^	72.81^c^	3.98^ab^	3.90^f^
6F		12.5	0.954^b^	0.998^b^	67.94^b^	71.14^bc^	3.89^c^	3.50^e^
SEM			0.0068	0.0019	1.141	1.350	0.081	0.042
Main effect								
Crude protein (g/kg)								
210			0.942	0.995	63.66	67.28	2.89	2.65
190			0.941	0.993	67.33	71.05	3.14	3.07
170			0.951	0.995	68.71	71.98	3.94	3.70
Energy (MJ/kg)								
13.0			0.944	0.993	67.60	71.18	3.32	3.22
12.5			0.946	0.995	65.53	69.02	3.32	3.06
Significance								
Crude protein			0.252	0.360	<0.001	0.004	<0.001	<0.001
Energy			0.746	0.166	0.034	0.059	0.936	<0.001
Crude protein × energy			0.047	0.009	0.013	0.007	0.014	<0.001
7G (150 g/kg CM with 190 g/kg CP)			0.959	0.995	60.01	62.29	3.19	2.98
Pair-wise comparisons (*P*-value)								
2B vs. 7G			0.002	0.059	<0.001	<0.001	0.736	0.005

a,b,c Means within columns not sharing a common superscript are significantly different at the 5% level of probability.

**Table 10:** The effects of dietary treatments on apparent distal ileal digestibility coefficients of essential amino acids at 36 d posthatch.

Diet	Treatment		Arginine	Histidine	Isoleucine	Leucine	Lysine	Methionine			
	Crude protein (g/kg)	Energy (MJ/kg)							Phenylalanine	Threonine	Valine
1A	210	13.0	0.911	0.853	0.869	0.864	0.882	0.943	0.889	0.842	0.856
4D		12.5	0.887	0.816	0.857	0.849	0.873	0.948	0.875	0.829	0.843
2B	190	13.0	0.884	0.809	0.855	0.852	0.848	0.935	0.881	0.822	0.845
5E		12.5	0.889	0.804	0.883	0.878	0.875	0.956	0.899	0.850	0.867
3C	170	13.0	0.884	0.792	0.872	0.868	0.864	0.951	0.893	0.835	0.858
6F		12.5	0.892	0.809	0.898	0.894	0.891	0.963	0.909	0.860	0.882
SEM			0.0075	0.0125	0.0102	0.0105	0.0111	0.0055	0.0081	0.0107	0.0102
Main effect											
Crude protein (g/kg)											
210			0.899	0.834^b^	0.863	0.856	0.878	0.946	0.882	0.836	0.850
190			0.887	0.807^a^	0.869	0.865	0.862	0.945	0.890	0.836	0.856
170			0.888	0.801^a^	0.885	0.856	0.877	0.957	0.901	0.847	0.870
Energy (MJ/kg)											
13.0			0.893	0.818	0.865	0.861	0.865	0.943^a^	0.888	0.833	0.853
12.5			0.889	0.809	0.879	0.874	0.880	0.956^b^	0.894	0.846	0.864
Significance											
Crude protein			0.194	0.026	0.099	0.075	0.261	0.076	0.084	0.468	0.139
Energy			0.557	0.397	0.092	0.153	0.105	0.007	0.339	0.135	0.194
Crude protein × energy			0.074	0.120	0.108	0.103	0.188	0.322	0.101	0.122	0.138
7G (150 g/kg CM with 190 g/kg CP)			0.901	0.819	0.862	0.864	0.864	0.955	0.891	0.822	0.845
Pair-wise comparisons (*P*-value)											
2B vs. 7G			0.114	0.564	0.580	0.393	0.290	0.017	0.634	0.958	0.970

a,b,c Means within columns not sharing a common superscript are significantly different at the 5% level of probability.

**Table 11:** The effects of dietary treatments on apparent distal ileal digestibility coefficients of non-essential amino acids at 36 d posthatch.

Diet	Treatment		Alanine	Aspartic acid	Glutamic acid	Glycine	Proline	Serine	Tyrosine
	Crude protein (g/kg)	Energy (MJ/kg)							
1A	210	13.0	0.787	0.814	0.922	0.821	0.909	0.854	0.878
4D		12.5	0.774	0.791	0.912	0.792	0.897	0.835	0.864
2B	190	13.0	0.683	0.701	0.910	0.834	0.900	0.800	0.862
5E		12.5	0.752	0.756	0.914	0.830	0.897	0.824	0.880
3C	170	13.0	0.645	0.603	0.895	0.849	0.880	0.759	0.864
6F		12.5	0.712	0.694	0.901	0.850	0.878	0.796	0.887
Main effect									
Crude protein (g/kg)									
210			0.780^b^	0.802^c^	0.917^b^	0.807^a^	0.903	0.844^c^	0.871
190			0.718^a^	0.729^b^	0.912^b^	0.832^b^	0.899	0.812^b^	0.871
170			0.678^a^	0.649^a^	0.898^a^	0.849^b^	0.879	0.777^a^	0.876
Energy (MJ/kg)									
13.0			0.705^a^	0.706^a^	0.909	0.835	0.891	0.804	0.868
12.5			0.746^b^	0.747^b^	0.909	0.824	0.896	0.818	0.877
Significance									
Crude protein			0.001	<0.001	0.029	<0.001	<0.003	<0.001	0.857
Energy			0.035	0.041	0.991	0.183	0.302	0.217	0.249
Crude protein × energy			0.147	0.062	0.484	0.239	0.696	0.113	0.131
P7G (150 g/kg CM with 190 g/kg CP)			0.750	0.731	0.906	0.831	0.870	0.796	0.870
PPair-wise comparisons (*P*-value)									
P2B vs. 7G			0.038	0.336	0.692	0.874	0.004	0.826	0.530

a,b,c Means within columns not sharing a common superscript are significantly different at the 5% level of probability.

**Table 12:** The effects of dietary crude protein concentrations on amino acid and ammonia concentrations (µg/mL) in systemic plasma in broiler chickens at 34 d posthatch.

Dietary CP (g/kg)	Arg	His	Ile	Leu	Lys	Met	Phe	Thr	Trp	Val
210	40.5	5.37	16.1	16.9	26.1	16.1^a^	17.9	89.7^a^	3.76	28.0
190	37.6	4.08	12.7	16.0	24.7	23.1^b^	19.5	103.0^a^	3.20	26.2
170	35.9	4.24	12.5	15.4	37.4	28.2^c^	20.5	123.4^b^	3.14	26.9
SEM	5.10	0.663	1.13	1.52	4.93	1.47	1.00	6.07	0.254	1.91
*P*-value	0.817	0.350	0.072	0.784	0.173	<0.001	0.210	0.005	0.193	0.799
Linear effects										
r =	0.161	0.291	0.485	0.177	−0.371	−0.831	−0.431	−0.709	0.402	0.107
*P*-value	0.523	0.242	0.042	0.483	0.130	<0.001	0.074	0.001	0.099	0.672


Dietary CP (g/kg)	Ala	Asn	Asp	Cys	Glu	Gln	Gly	Pro	Ser	Tyr	NH_4_OH
210	63.7^b^	31.4^c^	3.61^b^	13.7^b^	18.1	275.0	38.4^a^	64.7	53.5	37.8^a^	1.87^a^
190	55.8^b^	23.6^b^	3.13^b^	12.8^b^	16.6	303.0	56.1^b^	67.5	52.0	45.2^b^	2.04^ab^
170	41.1^a^	17.0^a^	2.46^a^	11.0^a^	16.1	252.1	68.7^b^	48.8	59.5	52.6^c^	2.19^b^
SEM	4.03	2.00	0.209	0.53	1.06	24.7	4.75	5.57	4.56	2.31	0.082
*P*-value	0.004	0.001	0.005	0.009	0.401	0.370	0.002	0.066	0.484	0.002	0.040
Linear effects											
r =	0.711	0.797	0.710	0.672	0.324	0.158	−0.757	0.435	−0.230	−0.760	−0.607
*P*-value	0.001	<0.001	0.001	0.002	0.190	0.531	<0.001	0.072	0.359	<0.001	0.010

a,b,c Means within columns not sharing a common superscript are significantly different at the 5% level of probability.

The 0.5 MJ reduction in energy density in 170 g/kg CP diets compromised FCR by 3.25% (1.525 vs. 1.477), which was significant (*P* = 0.013) based on a pair-wise comparison. The energy density reduction had lesser impacts on FCR in birds offered 210 and 190 g/kg CP diets. This outcome does not support the hypothesis that reduced-CP diets with lower energy densities can support acceptable growth performance.

In the present study, the CP reduction from 210 to 170 g/kg increased AME by 0.37 MJ (14.99 vs. 14.62 MJ/kg) and AMEn by 0.67 MJ (13.75 vs. 13.08 MJ/kg) in 13.0 MJ/kg energy density diets. This is a similar response to that observed in a series of studies with maize-based diets (Chrystal et al., 2020a,b,c; 2001), where AME was increased by 0.34 MJ (13.16 vs. 12.82 MJ/kg) and AMEn by 0.43 MJ (12.21 vs. 11.78 MJ/kg) following an average CP reduction from 210 to 163 g/kg. However, the validity of these improvements in energy utilization is open to question. For instance, contradictory outcomes were observed in 4 studies (Greenhalgh et al., 2020b; Yin et al., 2020; Chrystal et al., 2021; Greenhalgh et al., 2022a) in which energy uplifts were not observed in reduced-CP, wheat-based diets. Collectively, an average CP reduction from 214 to 168 g/kg depressed AME by 0.22 MJ (12.10 vs. 12.32 MJ/kg) in these 4 studies. The compositions of standard and reduced-CP broiler diets are quite different in terms of protein, starch and lipid which is reflected in the present study in that GE of the 7 experimental diets ranged from 15.66 to 16.63 MJ/kg; moreover, Mateos et al. (2019) contended that the energy density of different feed ingredients may not be additive. Also, AME and AMEn are determined on a total tract basis and may be confounded by the impacts of hind-gut fermentation (Qaisrani et al., 2015). Thus, the notional improvements in energy utilization should be treated with caution. In the present study, dietary CP reductions linearly increased N-retention, which is a common observation. There were increases in plasma-NH_3_ concentrations; however, plasma-NH_3_ concentrations are essentially derived from the postenteral deamination of surplus amino acids (Brosnan, 2003); whereas, N-retention is a total tract assessment.

A complicating factor in the present study was that dietary energy density reductions were achieved by manipulating dietary inclusions of soy oil, maize starch and wheat (Table 2). A multiple linear regression (r = 0.670; *P* < 0.001) was deduced to determine the impact of dietary inclusions of three feed ingredients on AME where it is revealed the significant effects of these feed ingredients on AME, as demonstrated by following equation:

$$\text{AM}E_{\left(\text{MJ}/\text{kg}\right)}=8.56+0.046*\text{soy}\;\text{oi}l_{\left(g/\text{kg}\right)}+0.010*\text{maize}\;\text{starc}h_{\left(g/\text{kg}\right)}+0.006*\text{whea}t_{\left(g/\text{kg}\right)}.$$

**Figure 1:**
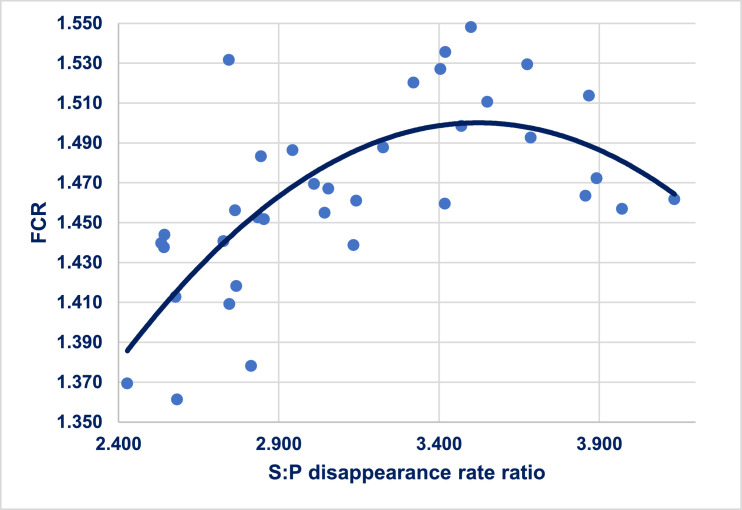
Quadratic relationship (r = 0.715; *P* < 0.0001) between distal ileal starch:protein disappearance rate ratio and FCR where y = 0.318 + 0.671*ratio – 0.095*ratio^2^.

A quadratic relationship (r = 0.715; *P* < 0.001) was detected between starch:protein disappearance rate ratios and FCR as shown in Figure 1. Efficiency of feed conversion improved as disappearance rate ratios condensed below 3.54, which corresponded to the maximum FCR of 1.505. Unsurprisingly, analyzed dietary starch:protein ratios were aligned with the starch:protein disappearance rate ratios observed in birds; collectively, the analyzed ratios increased from 2.24 to 3.09 pursuant to dietary CP reductions from 210 to 170 g/kg (Table 4). This outcome emphasizes both the importance of starch:protein digestive dynamics in general terms (Liu and Selle, 2015) and also suggests that increased starch concentrations in reduced-CP diets may be deleterious. Interestingly, condensing or “capping” dietary starch:protein ratios has shown some promise in both wheat-based (Greenhalgh et al., 2020b) and maize-based (Greenhalgh et al., 2022b) reduced-CP diets.

Significant responses across ileal digestibility coefficients of essential amino acids were limited to a 1.38% increase in methionine following the energy density reduction and histidine digestibility was depressed by up to 3.96% by dietary CP reductions. Across the non-essential amino acids the dietary CP reductions significantly decreased digestibilities of alanine, aspartic acid, glutamic acid, proline and serine, but significantly increased glycine digestibility. That glycine increased may be attributed to non-protein bound glycine inclusions across all dietary treatments as NPBAA are notionally completely digestible. Alternatively, dietary concentrations of the 5 amino acids with decreased digestibilities declined following dietary CP reductions as they were not supplemented with nonbound entities. Thus, the likelihood is that their concentrations of dietary origin in ileal digesta were diluted by endogenous and microbial amino acids which would depress apparent ileal digestibility coefficients.

Dietary CP reductions linearly associated with increased (r = −0.709; *P* = 0.001) free threonine plasma concentrations by 37.6% in the present study. This is a relatively modest increase given a 2-fold increase (179.6 vs. 89.6 µg/mL) in threonine concentrations was observed in birds offered wheat-based diets following the CP reduction from 222 to 165 g/kg in Chrystal et al. (2021). The growth performance of birds offered 170 g/kg CP diets in the present study was very much better than their 165 g/kg CP counterparts in Chrystal et al. (2021), and it is possible that free plasma threonine concentrations can serve as biomarkers for the correctness with which reduced-CP diets are formulated (Macelline et al., 2021). The threonine increases may stem from the down regulation of threonine-3-dehydrogenase activity in the liver which converts threonine to glycine and acetyl-CoA (Akagi et al., 2004). It is possible that acetyl-CoA derived from high starch concentrations in reduced-CP diets inhibited threonine-3-dehydrogenase activity via negative feed-back mechanisms (Guerranti et al., 2001).

It is instructive to compare the outcomes of birds offered diet 2B (37.4 g/kg soybean meal) and 7G (150 g/kg canola meal) as there was no significant differences in growth performance while this was not a direct comparison, the replacement of soybean meal with canola meal in 190 g/kg CP diets displayed some promise. Clearly, further research into canola meal inclusions in reduced-CP broiler diets is warranted, especially under Australian conditions. The substitution of soybean meal with canola meal will condense dietary starch:protein ratios and may depress total nonbound amino acid inclusions, both of which should prove advantageous.

## CONCLUSIONS

Three tiers of CP reductions in wheat-based broiler diets with 2 energy densities linearly associated with compromised growth performance, including weight gain (r = −0.783; *P* < 0.001), feed intake (r = −0.549; *P* = 0.001), and FCR (r = 0.682; *P* < 0.001). This outcome supports the contention that the successful development of particularly wheat-based, reduced-CP diets constitutes a definite challenge. Reducing dietary energy density by 0.5 MJ significantly compromised FCR in birds offered 170 g/kg CP diets, although dietary CP reductions uplifted energy utilization. It does appear that determinations of energy utilization (AMEn more so than AME) in broilers offered diets with a range of CP levels are subject to aberrations, which may partially stem from substantial differences in their composition and a lack of additivity of the energy contributions from the various dietary components. The relevance of digestive dynamics was in evidence as narrowing ileal starch:protein disappearance rate ratios improved FCR in a quadratic manner. The inclusion of canola meal at 150 g/kg at the expense of soybean meal displayed promise in 190 g/kg CP diets, although the comparison could have been more direct. Finally, the implications of the significant relationship between dietary NPBAA inclusions and elevated plasma NH_3_ concentrations are profound for the successful development of reduced-CP diets.

## References

[bib0001] ABARES (2023).

[bib0002] Akagi S., Sato K., Ohmori S. (2004). Threonine metabolism in Japanese quail liver. Amino Acids.

[bib0003] Bach Knudsen K.E. (1997). Carbohydrate and lignin contents of plant materials used in animal feeding. Anim. Feed Sci. Technol..

[bib0004] Baker D.H. (2009). Advances in protein-amino acid nutrition in poultry. Amino Acids.

[bib0005] Brosnan J.T. (2003). Interorgan amino acid transport and its regulation. J. Nutr..

[bib0006] Bryden W.L., Li X., Ravindran G., Hew L.I., Ravindran V. (2009).

[bib0007] Chrystal P.V., Greenhalgh S., McInerney B.V., McQuade L.R., Akter Y Y., de Paula Dorigam J.C., Selle PH P.H., Liu S.Y. (2021). Maize-based diets are more conducive to crude protein reductions than wheat-based diets for broiler chickens. Anim. Feed Sci. Technol..

[bib0008] Chrystal P.V., Moss A.F., Khoddami A., Naranjo V.D., Selle P.H., Liu S.Y. (2020). Effects of reduced crude protein levels, dietary electrolyte balance, and energy density on the performance of broiler chickens offered maize-based diets with evaluations of starch, protein, and amino acid metabolism. Poult. Sci..

[bib0009] Chrystal P.V., Moss A.F., Khoddami A., Naranjo V.D., Selle P.H., Liu S.Y. (2020). Impacts of reduced-crude protein diets on key parameters in male broiler chickens offered maize-based diets. Poult. Sci..

[bib0010] Chrystal P.V., Moss A.F., Yin D., Khoddami A., Naranjo V.D., Selle P.H., Liu S.Y. (2020). Glycine equivalent and threonine inclusions in reduced-crude protein, maize-based diets impact on growth performance, fat deposition, starch-protein digestive dynamics and amino acid metabolism in broiler chickens. Anim. Feed Sci. Technol..

[bib0011] Giuberti G., Gallo A., Cerioli C., Masoero F. (2012). In vitro starch digestion and predicted glycemic index of cereal grains commonly utilized in pig nutrition. Anim. Feed Sci. Technol..

[bib0012] Greenhalgh S., Chrystal P.V., Lemme A., de Dorigam J.C.P., Macelline S.P., Liu S.Y., Selle P.H. (2022). Capping dietary starch:protein ratios enhances performance of broiler chickens offered reduced-crude protein, maize-based diets. Anim. Feed Sci. Technol..

[bib0013] Greenhalgh S., Chrystal P.V., Selle P.H., Liu S.Y. (2020). Reduced-crude protein diets in chicken-meat production: justification for an imperative. Worlds Poult. Sci. J..

[bib0014] Greenhalgh S., Lemme A., de Dorigam J.C.P., Chrystal P.V., Macelline S.P., Liu S.Y, Selle P.H. (2022). Dietary crude protein concentrations, feed grains, and whey protein interactively influence apparent digestibility coefficients of amino acids, protein, starch, and performance of broiler chickens. Poult. Sci..

[bib0015] Greenhalgh S., McInerney B.V., McQuade L.R., Chrystal P.V., Khoddami A., Zhuang M.A.M., Liu S.Y., Selle P.H. (2020). Capping dietary starch:protein ratios in moderately reduced crude protein, wheat-based diets showed promise but further reductions generated inferior growth performance in broiler chickens from 7 to 35 days post-hatch. Anim. Nutr..

[bib0016] Guerranti R., Pagani R., Neri S., Errico S.V., Leoncini R., Marinello E. (2001). Inhibition and regulation of rat liver L-threonine dehydrogenase by different fatty acids and their derivatives. Biochim. Biophys. Acta.

[bib0017] Hill F.W., Anderson D.L. (1958). Comparison of metabolizable energy and productive energy determinations with growing chicks. J. Nutr..

[bib0018] Liu S.Y., Selle P.H. (2015). A consideration of starch and protein digestive dynamics in chicken-meat production. Worlds Poult. Sci. J..

[bib0019] Macelline S.P., Chrystal P.V., Liu S.Y., Selle P.H. (2021). Implications of elevated threonine plasma concentrations in the development of reduced-crude protein diets for broiler chickens. Anim. Prod. Sci..

[bib0020] Mahasukhonthachat K., Sopade P.A., Gidley M.J. (2010). Kinetics of starch digestion and functional properties of twin-screw extruded sorghum. J. Cereal Sci..

[bib0021] Mateos G.G., Cámara L., Fondevila G., Lázaro R.P (2019). Critical review of the procedures used for estimation of the energy content of diets and ingredients in poultry. J. Appl. Poult. Res..

[bib0022] Musharaf N.A., Latshaw I.D (1999). Heat increment as affected by protein and amino acid nutrition. Worlds Poult. Sci. J..

[bib0023] Namroud N.F., Shivazad M., Zaghari M. (2008). Effects of fortifying low crude protein diet with crystalline amino acids on performance, blood ammonia level, and excreta characteristics of broiler chicks. Poult. Sci..

[bib0024] Ospina-Rojas I.C., Murakami A.E., Duarte C.R.A, Eyng C., Oliveira A.L, Janeiro V. (2014). Valine, isoleucine, arginine and glycine supplementation of low-protein diets for broiler chickens during the starter and grower phases. Br. Poult. Sci..

[bib0025] Ospina-Rojas I.C., Murakami A.E., Moreira I., Picoli K.P., Rodrigueiro R.J.B., Furlan A.C. (2013). Dietary glycine+serine responses of male broilers given low-protein diets with different concentrations of threonine. Brit. Poult. Sci..

[bib0026] Qaisrani S.N., van Krimpen M.M., Kwakkel R.P., Verstegen M.W.A., Hendriks W.H. (2015). Dietary factors affecting hindgut protein fermentation in broilers: a review. Worlds Poult. Sci. J..

[bib0027] Salway J.G. (2018). The Krebs uric acid cycle: a forgotten Krebs cycle. Trends Biochem. Sci..

[bib0028] Selle P.H., Macelline S.P., Chrystal P.V., Liu S.Y. (2023). The challenge to reduce crude protein contents of wheat-based broiler diets. Anim. Prod. Sci..

[bib0029] Selle P.H., Macelline S.P., Chrystal P.V., Liu S.Y. (2023). Amino acid imbalances. Proc. Aust. Poult. Sci. Symp..

[bib0030] Selle P.H., Macelline S.P., Greenhalgh S., Chrystal P.V., Liu S.Y. (2022). Identifying the shortfalls of crude protein-reduced, wheat-based broiler diets. Anim. Nutr..

[bib0031] Selle P.H., Truong H.H., McQuade L.R., Moss A.F., Liu S.Y. (2016). Reducing agent and exogenous protease additions, individually and in combination, to wheat-and sorghum-based diets interactively influence parameters of nutrient utilisation and digestive dynamics in broiler chickens. Anim. Nutr..

[bib0032] Siriwan P., Bryden W.L., Annison E.F (1994). Use of guanidinated dietary protein to measure losses of endogenous amino acids in poultry. Br. J. Nutr..

[bib0033] Yin D., Chrystal P.V., Moss A.F., Liu S.Y., Selle P.H. (2020). Effects of reduced crude protein and whole grain feeding on performance and amino acid metabolism in broiler chickens offered wheat-based diets. Anim. Feed Sci. Technol..

